# Uneventful Coadministration of Seasonal Influenza and COVID-19 BNT162b2 Vaccines Two Weeks Post-Influenza Vaccination in an Egg-Allergic Subject: A Case Report

**DOI:** 10.3390/vaccines11050950

**Published:** 2023-05-05

**Authors:** Anwar A. Sayed

**Affiliations:** Department of Medical Microbiology and Immunology, College of Medicine, Taibah University, Madinah 42353, Saudi Arabia; dsayed@taibahu.edu.sa

**Keywords:** COVID-19, double vaccination, egg allergy, influenza, vaccine safety

## Abstract

The COVID-19 pandemic took the world by storm, and although it has taken the world’s attention, it did not stop the spread of other communicable diseases. Seasonal influenza is a viral infection that could cause severe disease; therefore, annual influenza vaccination is highly recommended, especially among patients with a weakened immune system. However, such vaccination is contraindicated for people with hypersensitivity to the vaccine or any of its components, e.g., eggs. This paper describes a case of an egg-allergic individual who received an influenza vaccine containing egg protein, which only caused mild tenderness at the site of injection. Two weeks later, the subject received a double vaccination of a second booster dose of Pfizer-BioNTech and the seasonal influenza vaccine. The patient reported no local or systemic adverse reactions to the vaccine. This case report suggests vaccination safety for subjects with mild allergies to vaccine components.

## 1. Introduction

The COVID-19 pandemic took the world by storm, and although it has taken the world’s attention, it did not stop the spread of other communicable diseases, such as seasonal influenza. Seasonal influenza is a viral infection that could cause severe illness, especially among patients with a weakened immune system, e.g., elderly patients, leading to hospitalisation [1,2]. Therefore, public health authorities recommend the uptake of seasonal influenza vaccines annually to protect against severe cases of influenza infections. The uptake of influenza vaccines should not negate or negatively affect COVID-19 vaccinations, and it is recommended that the population, especially those at risk of severe infections, should be vaccinated against both pathogens [3,4]. The full dose of COVID-19 vaccination was one of the instrumental measures taken by the Saudi public health authorities to tackle the pandemic [5,6,7,8].

Vaccines are generally safe to administer but are contraindicated to those with severe allergies against the vaccine or one of its components. For example, seasonal influenza vaccines are usually raised in eggs; hence, they are contraindicated in people with an egg allergy [9]. However, not all allergies are at the same level of severity, and the ratio of the benefit (of vaccination) to the risk (of having an allergic reaction) should be carefully weighed to determine whether to vaccinate subjects or not [10].

## 2. Case Presentation

This is a case of a 34-year-old male, a non-smoker, with a body mass index (BMI) of 49.8 and a known case of egg allergy. The patient described his allergy to eggs as mild, in which he develops urticaria upon consumption of eggs across several consecutive days. The patient previously received two doses of Adenovector AstraZeneca COVID-19 vaccine and a booster dose of Pfizer-BioNTech.

The patient received a dose of the quadrivalent influenza vaccine (Vaxigrip Tetra^®^) (day 0). According to the manufacturer’s instructions, the vaccine is contraindicated for people with hypersensitivity to eggs (ovalbumin, chicken proteins). On day 14, the patient received a COVID-19 booster dose and was offered a dose of the influenza vaccine, to which he agreed. The patient received another shot of the influenza vaccine and the second booster dose of the COVID-19 vaccine (Pfizer-BioNTech), in the left and right arm, respectively. The two shots of the influenza vaccine the patient received were of the same type and injection site (right arm).

**Figure 1:**
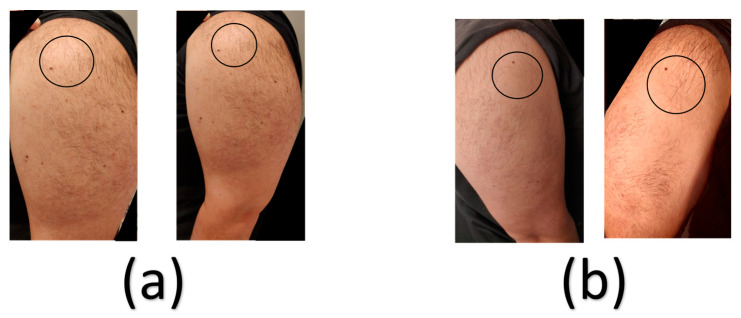
Sites of both the COVID-19 and influenza vaccines’ injection sites. Images in panel (**a**) show the injection site of the COVID-19 vaccine in the left arm (circled in black). Images in panel (**b**) show the injection site of the influenza vaccine in the right arm (circled in black).

The patient reported local tenderness around the injection site following the first influenza vaccine on day 0; however, no systematic manifestations were reported, such as fever, shivering, fatigue, or body aches. Upon the double vaccination on day 14, the patient was kept under medical surveillance in a healthcare facility for 30 min as a precautionary measure, during which the patient did not develop any adverse reactions. Following the double vaccination, the patient did not develop any significant local reactions, e.g., severe redness, pain, or tenderness. However, a localised rash can be observed at the site of the influenza vaccine injection (Figure 1).

Similarly, no systemic vaccine adverse reactions were observed, and the patient did not report taking any over-the-counter analgesics/antipyretics, e.g., Paracetamol or Ibuprofen. The patient was invited for a blood investigation three days after the double vaccination. The patient’s haematological parameters were all within the reference range, except for the mean corpuscular volume (MCV), which was 0.1 fL below the reference range. The patient’s Food Allergen Test showed a very minor positivity to alpha-lactalbumin and positivity to shrimp/prawn (Table 1).

## 3. Discussion

Allergy, or hypersensitivity, can be classified into immunoglobulin (Ig)E-mediated and non-IgE-mediated disease. IgE-mediated hypersensitivity shows rapid clinical features, such as urticaria and/or angioedema, which could further develop into a life-threatening anaphylactic reaction [11]. Such allergies could possibly be diagnosed by careful history taking and commercially available allergen-specific antibodies. Some non-IgE-mediated hypersensitivities, such as delayed hypersensitivities, could take days before clinical manifestations appear. These delayed hypersensitivities are partially attributed to cellular (T cell) response, and reaching such a diagnosis is challenging with commercially available tools, such as the skin prick test and antibody testing [12]. The patient, presented in this case study, seemed to have a delayed hypersensitivity to eggs, as evident from his account of his allergy (history taking), the lack of immediate manifestations developed after vaccinations, and the allergen test result showing a lack of egg-specific IgE.

Interestingly, the practice of mass vaccination was concurrent with the increase in the prevalence of allergy among children, leading researchers to hypothesise a link between vaccination and allergy [13,14]. However, Navaratna and colleagues attempted to evaluate such a link, which was found to be non-evident using a systematic review and meta-analysis of 35 cohort studies and 7 randomised controlled trials [15].

The safety of vaccinating subjects with egg allergies and egg-based vaccines has always been of concern to public health policymakers. In Denmark, a retrospective study was conducted on 32 patients with egg allergy who were vaccinated with an egg-based vaccine, the measles, mumps, and rubella (MMR) vaccine [16]. Andersen and Jørgensen found that the MMR vaccinations were uneventful, given that the first and second doses of the MMR vaccine were over two years apart. In 2010, Gagnon and colleagues evaluated the safety of the egg-based H1N1 adjuvant vaccine in patients with egg allergy. After receiving either a single or double dose of the vaccine, they demonstrated that less than 2% and 15% reported signs or symptoms of allergic reactions at 1 and 24 h post-vaccination, respectively [17]. Similarly, the presented case reported local adverse reactions, namely tenderness following the first dose and rash after the second dose, in the first 24 h post-vaccination.

Although theoretically safe, the coadministration of vaccines was previously assessed to ensure public safety. In a retrospective cohort study, Hause and colleagues reported the safety of simultaneous administration of COVID-19 mRNA booster dose and influenza vaccines in over 92 thousand persons in the US [18]. They reported that the dual vaccination significantly increased the likelihood of immunisation adverse reactions, both local and systemic, compared with administering the COVID-19 booster dose alone. Similarly, Lazarus and colleagues reported the findings of their clinical trial in the UK on the safety and immunogenicity of a double vaccination of COVID-19 (ChAdOx1 or BNT162b2) and influenza vaccination, regardless of their preparation methods [19]. They found that double vaccination with COVID-19 and influenza vaccines raises no safety concerns, increases vaccination coverage, and reduces the burden on healthcare services. The Centers for Disease Control and Prevention (CDC) recommended that the coadministration of influenza vaccine with any other vaccine, e.g., COVID-19, should be administered in different anatomic sites [20], which was followed in the presented case. However, it only limited its contraindications for those with severe allergic reactions to any of the vaccine components.

The patient, despite his mild allergy to eggs, has requested to take the flu vaccine. As a subject with a high risk of developing an allergic or even anaphylactic response, special measures should be taken prior to vaccinating such a subject. Careful history should be taken to detail the egg allergy, in terms of the required quantity to trigger the allergic response and the nature of the allergic response, i.e., the time required for symptoms to appear and the extent of these symptoms. Vaccination should also take place at a healthcare facility equipped to deal with an immediate anaphylactic reaction, e.g., the presence of different methods of epinephrine administration (intramuscular and intravenous (IV) infusion), oxygen supply, and IV fluid supply [21]. The vaccinated subject should be put under observation for 30 min after vaccination to monitor the appearance of any allergic symptoms and the subject’s general condition. In case of vaccination at a primary healthcare centre or a non-medical facility, quick access to a nearby healthcare facility should be readily available in the form of a well-equipped ambulance vehicle.

Wang et al. reported a case of delayed skin rash that resolved spontaneously after vaccination with Moderna’s mRNA-1273 COVID-19 vaccine [22]. Interestingly, the presented case developed a similar localised rash, but milder, in the right arm, the one that received the influenza vaccine.

Although vaccines were invaluable in tackling and limiting the impact of the COVID-19 pandemic, several studies in Saudi Arabia have documented widespread hesitance and resistance to COVID-19 vaccinations [23,24]. Similar to studies from other countries, such an attitude seems to originate from the mass misinformation about some harmful vaccine components presented on social media, as well as in other sources of information [25]. Hence, the presence of an allergy, e.g., egg allergy, might be considered a precipitating factor to such vaccine hesitance, at a time when we are still tackling COVID-19 infection at a global scale. This could also cause parents to refrain from vaccinating their children due to some perceived harm [26], which may not be accurate.

As the present study is a case report, it carries similar limitations present in other clinical case reports. The study presents unusual findings in a single subject, making it lacking any epidemiological quantities, such as prevalence or risk ratios. Additionally, the findings presented in this case report cannot be generalised to any particular population [27], whether a condition-based population (e.g., subjects with egg allergy) or a geographical-based population (e.g., Saudi population). Despite such a limitation, case studies should not be entirely dismissed, as they constitute the first level of evidence used to guide our evidence-based medical practice [28]. As expected when interpreting case reports, the findings presented here should be carefully considered and not be overinterpreted, i.e., tending towards generalising the results in the absence of statistical justification [29]. Lastly, it is important to note that the positive findings in this case report are not claimed to be caused by either one or both vaccinations. For example, the development of rash at the site of injection cannot be claimed to be caused by vaccination. Causality cannot be determined without the presence of a valid control, and hence, case studies are unable to demonstrate causality [30].

This case study presents a learning opportunity to medical practitioners, public health researchers, as well as the public. It paves the way for future studies to be conducted on the safety of receiving COVID-19 and influenza concomitantly, which would widen the coverage of both vaccines. The study also sheds light on revising the safety of influenza vaccines to subjects with egg allergy, and whether egg allergy should be an absolute contraindication to influenza vaccination.

## 4. Conclusions

Vaccinations are one of the public health cornerstones due to their paramount role in reducing communicable diseases and their safety profile. Except for live-attenuated vaccines, vaccines are generally safe to administer to everyone except those with an allergy to the vaccine or one of its components. Careful history taking should be carried out to determine the presence of an allergy before vaccination. More importantly, history taking should also differentiate the type of allergy and whether vaccination would pose an imminent risk to the subject. This case report provides a unique learning opportunity to reconsider what constitutes a valid contraindication to immunisation. It also paves the way for future studies to assess the safety of the egg-based vaccine for subjects with an egg allergy and its safety to be coadministered with COVID-19 vaccines.

## Figures and Tables

**Table 1:** Laboratory investigations three days after the coadministration of influenza and COVID-19 BNT162b2 vaccines.

Investigation	Result	Unit	Reference Range
Complete Blood Count			
Haemoglobin	14.93	g/dL	13.5–18.0
Haematocrit	42.80	%	40.0–54.0
RBC count	5.50	10^12^/L	4.50–6.10
MCV	**77.90**	fL	78.0–99.0
MCH	27.10	pg	27.0–32.0
MCHC	34.90	g/dL	32.0–36.0
RDW	14.10	%	11.5–14.5
WBC count	8.58	10^9^/L	4.0–10.5
Platelet count	225.10	10^9^/L	150–450
WBC Differential Count			
Neutrophils	56.64	%	40.0–80.0
Lymphocytes	34.35	%	20.0–40.0
Monocytes	6.12	%	2.0–10.0
Eosinophils	2.40	%	1.0–6.0
Basophils	0.48	%	0.0–2.0
Food Allergen Test			
Milk	Class 0.0 Negative *		Negative
Alpha-lactalbumin	Class 0.2 Negative		Negative
Beta-lactalbumin	Class 0.0 Negative		Negative
Casein	Class 0.2 Negative		Negative
Egg white	Class 0.0 Negative		Negative
Egg yolk	Class 0.0 Negative		Negative
Wheat flour	Class 0.0 Negative		Negative
Mango	Class 0.0 Negative		Negative
Orange	Class 0.0 Negative		Negative
Carrot	Class 0.0 Negative		Negative
Strawberry	Class 0.0 Negative		Negative
Shrimp/prawn	Class 1.7 Positive		Negative
Codfish	Class 0.0 Negative		Negative
Mussel	Class 0.0 Negative		Negative
Banana	Class 0.0 Negative		Negative
Cacao	Class 0.0 Negative		Negative
Onion	Class 0.0 Negative		Negative
Fig	Class 0.0 Negative		Negative
Tomato	Class 0.0 Negative		Negative
Date	Class 0.0 Negative		Negative
Kiwi	Class 0.0 Negative		Negative
Honey	Class 0.0 Negative		Negative
Soya bean	Class 0.0 Negative		Negative
Peanut	Class 0.0 Negative		Negative
Cashew nut	Class 0.0 Negative		Negative
Pecan nut	Class 0.0 Negative		Negative
Walnut	Class 0.0 Negative		Negative
Sesame seed	Class 0.0 Negative		Negative
Cheese mix	Class 0.0 Negative		Negative

Results presented in bold are the ones higher/lower than the reference range. * Class 0: no detection of specific IgE abs, Class 1: low concentration of specific IgE abs, Class 2: moderately elevated concentration of specific IgE abs, Class 3: significantly elevated concentration of specific IgE abs, Class 4: high concentration of specific IgE abs, Class 5: very high concentration of specific IgE abs, Class 6: extremely high concentration of specific IgE abs.3.3. Formatting of mathematical components.

## Data Availability

All relevant data are presented in the manuscript.
